# More than Decoration: Roles for Natural Killer Group 2 Member D Ligand Expression by Immune Cells

**DOI:** 10.3389/fimmu.2018.00231

**Published:** 2018-02-12

**Authors:** Andrew P. Trembath, Mary A. Markiewicz

**Affiliations:** ^1^Department of Microbiology, Molecular Genetics and Immunology, University of Kansas Medical Center, Kansas City, MO, United States

**Keywords:** natural killer group 2 member D ligands, natural killer group 2 member D, immune cells, immune regulation, natural killer cells, T cells

## Abstract

The activating immune receptor natural killer group 2 member D (NKG2D), which is expressed by natural killer cells and T cell subsets, recognizes a number of ligands expressed by “stressed” or damaged cells. NKG2D has been extensively studied for its role in tumor immunosurveillance and antiviral immunity. To date, the majority of studies have focused on NKG2D-mediated killing of target cells expressing NKG2D ligands. However, with a number of reports describing expression of NKG2D ligands by cells that are not generally considered stressed, it is becoming clear that some healthy cells also express NKG2D ligands. Expression of these ligands by cells within the skin, intestinal epithelium, and the immune system suggests other immune functions for NKG2D ligand expression in addition to its canonical role as a “kill me” signal. How NKG2D ligands function in this capacity is just now starting to be unraveled. In this review, we examine the expression of NKG2D ligands by immune cells and discuss current literature describing the effects of this expression on immunity and immune regulation.

## Introduction

The demands placed on the immune system are immense and highly complex. It is tasked with protecting the body against untold external threats while maintaining a balance between immune defense and autoimmune damage, the stakes are literally life and death. Fortunately, millions of years of evolution have resulted in immunological systems which are equally complex and necessarily efficient. Increasingly, we are coming to appreciate that few immune mechanisms are “single use,” with many systems having distinct functions dependent upon setting and context. While this immunological multipurposing leads to a capable and nuanced immune response, it puts the onus on us to tease out the different roles played by many immune system components. A prime example is presented in the activating immune receptor natural killer group 2 member D (NKG2D) and its ligands.

Natural killer group 2 member D, which is encoded by the gene *killer cell lectin-like receptor K1* (*Klrk1*) and designated CD314, is one of the best-studied activating immune receptors. NKG2D is expressed by all human and mouse natural killer (NK) cells, all human CD8^+^ T cells, activated mouse CD8^+^ T cells, NKT cells, subsets of γδ T cells, and rare CD4^+^ T cells in both human and mouse (1–4). NKG2D binds to a number of endogenous ligands that are induced by cellular stress and originally believed to be effectively absent from healthy cells (5, 6). There are eight known human NKG2D ligands. These are major histocompatibility complex (MHC) class I polypeptide-related sequence A (MICA) and B (MICB), and the retinoic acid early transcript 1 (RAET1) family of proteins, which are better known as the UL16-binding proteins (ULBP1-6). There are nine known ligands for NKG2D in mouse. They are RAE-1α-ε, H60a-c, and murine ULBP-like transcript 1 (MULT1), which are all orthologs of human RAET1. NKG2D ligands are all distantly related to MHC class I molecules, but do not associate with β2 microglobulin or bind peptide, and are tethered to the cell membrane *via* a GPI anchor or transmembrane domain (7). Specifically, MICA, MICB, ULBP4, H60a, H60b, and MULT1 have transmembrane domains, while ULBP1, ULBP3, and ULBP6, RAE-1α-ε, and H60c are attached to the cell surface *via* GPI anchors. Interestingly, ULBP2, ULBP5, and certain alleles of MICA can be associated with the membrane *via* a transmembrane domain or by GPI anchor (7, 8). Ligands can be shed from the cell surface *via* proteolytic cleavage, alternative splicing, phosphoinositide phospholipase C, or exosome release (9). While the ligands do have different binding affinities with NKG2D, all NKG2D ligands are believed to signal similarly through NKG2D (5, 6). NKG2D ligands have generally been considered markers of “altered self,” being induced by stress, such as cellular transformation or infection, and acting as a distress signal to target affected cells for immune killing.

Natural killer group 2 member D has been extensively studied for its involvement in antitumor surveillance and viral immunity, where it directs NK cell and CD8^+^ T cell recognition of NKG2D ligand-expressing cancerous or virally infected cells. NKG2D functions as a homodimer, with a short cytoplasmic tail that does not contain any signaling motifs. To signal, NKG2D associates with one of two adapter proteins, DNAX-activating protein of 10 kDa (DAP10) or DNAX-activating protein of 12 kDa (DAP12). In human and mouse T cells and NK cells, NKG2D associates with DAP10, which has a YINM motif that induces PI3 kinase and Grb2-Vav signaling (7, 10). In mouse NK cells, NKG2D also associates with DAP12, which is an immunotyrosine-based activation motif-bearing signaling molecule that signals through Syk and Zap70 (11–13). On NK cells, NKG2D is a primary activating receptor, triggering NK cell cytotoxicity and cytokine production in response to ligand-expressing cells. The function of NKG2D on CD8^+^ T cells is less well-defined with both co-stimulatory and T cell receptor-independent functions being described (1, 14–17). In addition to this well-studied role directing immune killing of ligand-expressing cells, a growing body of evidence suggests that NKG2D–NKG2D ligand interactions play other important roles in shaping the immune response. This idea came about after the appreciation of the importance of NKG2D ligand expression by otherwise healthy tissues (18). Numerous reports show expression of NKG2D ligands by healthy tissues, but until relatively recently, the effects of this NKG2D ligand expression were not explored in-depth. The expression of NKG2D ligands by healthy cells is the focus of a review by Eagle et al., wherein the authors address the potential significance of NKG2D ligand expression by both healthy hematopoietic and non-hematopoietic cells and discuss the need for more systematic study of the role of NKG2D–NKG2D ligand signaling in apparently healthy cells (18). In the years since this review, further evidence has accumulated that NKG2D ligand expression by healthy cells has distinct functions beyond targeting cells for immune killing. One major type of healthy cells, which evidence suggests routinely express NKG2D ligands, is cells of the hematopoietic lineage, specifically leukocytes. The role of NKG2D ligands in host defense as well as the mechanisms regulating ligand expression are discussed in detail elsewhere (6, 7). In this review, we focus on the expression of NKG2D ligands by immune cells and discuss what role this expression plays in the modulation of immune responses.

## Function of NKG2D Ligand Expression by T Cells

In their 1998 paper first describing the human NKG2D ligand MICA, Zwirner and colleagues showed that MICA was weakly expressed by freshly isolated CD4^+^ and CD8^+^ T cells, but that expression could be strongly induced in culture by addition of the polyclonal T cell activator phytohemagglutinin (19). Further investigation showed that MICA was induced on human T cells upon activation with anti-CD3 and anti-CD28 or PMA stimulation, and this induction could be inhibited in a dose-dependent manner by the NF-κB inhibitor sulfasalazine (20). In these studies, the authors suggest that MICA expression by T cells could participate in the maintenance of immune homeostasis through NKG2D-mediated NK cell killing of activated T cells (21). Indeed, a number of studies in both human and mouse have since observed expression of NKG2D ligands by activated T cells and found that this expression makes them susceptible to NKG2D-mediated killing. In mice, a study by Rabinovich et al. showed that upon activation, T cells from either C57BL/6 or Balb/c mice became susceptible to syngeneic killing by NK cells or lymphokine-activated killer cells (22). In Balb/c mice, this killing was mediated by NKG2D and was due to upregulation of an NKG2D ligand, most likely H60 (22). Curiously, however, no NKG2D ligands were detected on activated C57BL/6 T cells, suggesting that recognition and killing of activated syngeneic C57BL/6 T cells are mediated through a different receptor (22). In a model of graft-versus-host disease, Noval Rivas and colleagues found that transferred host-specific CD4^+^ T cells were limited by NKG2D-dependent killing by host NK cells (23). They found that upon antigen stimulation, monoclonal antigen-specific CD4^+^ T cells upregulated mRNA encoding the NKG2D ligands: MULT1 and H60. However, it should be noted that surface expression of MULT1 was not observed by flow cytometry, and surface expression of H60 proteins was not investigated (23). In humans, a similar finding was reported by Cerboni et al., who found that primarily MICA, but also ULBP1-3, was expressed by activated human CD4^+^ and CD8^+^ T cells upon antigen stimulation in an ataxia telangiectasia mutated/ataxia telangiectasia mutated- and Rad3-related protein (ATM)-dependent manner. In addition, expression of these ligands by activated T cells resulted in NKG2D-mediated NK cell lysis, again suggesting a potential mechanism for limiting T cell responses (24). Nielsen et al. also found that activated CD4^+^ T cells expressed MICA, MICB, and ULBP1-3 and were susceptible to NK cell lysis (25). Further evidence supporting this role comes from a recent study that showed expression of MICA and MICB by liver-infiltrating T cells in patients with chronic hepatitis B correlated with enhanced NK cell activation and NKG2D-dependent depletion of CD4^+^ T cells upon short-term *ex vivo* culture (26). However, it appears that NKG2D-mediated T cell killing does not always result in a reduced immune response. For instance, during *Mycobacterium tuberculosis* infection, NK cells were shown to control regulatory T cell (Treg) numbers through NKG2D-mediated lysis of NKG2D ligand-expressing Tregs (27).

As discussed earlier, multiple studies demonstrate that NKG2D ligand expression by human and murine T cells has an important function in regulating T cell responses by directing the elimination of activated T cells. However, there is also evidence of additional functions for NKG2D ligands expressed on apparently healthy T cells. Li et al. found that NKG2D ligands were expressed by double positive Balb/c thymocytes prior to fate determination, suggesting a role for NKG2D ligand expression in thymocyte development (28). It was also shown that human peripheral blood mononuclear cells (PBMCs), and in particular CD4^+^ T cells, can release soluble NKG2D ligands in response to superantigen stimulation. These soluble ligands were found to downregulate NKG2D expression by CD8^+^ T cells, which showed impaired proliferation, cytokine production, and cytotoxic activity (29). Finally, emerging evidence suggests that expression of NKG2D ligands by T cells directly affects the production of cytokines. A recent article reported that expression of ULBP proteins by CD4^+^ T cells from inflamed Crohn’s disease intestine positively correlated with release of IL-10, while the frequency of γδ^+^ and CD56^+^ cells expressing NKG2D negatively correlated with inflammation and pro-inflammatory cytokine release (30). Additionally, our laboratory recently demonstrated that T cells from non-obese diabetic (NOD) mice express H60a upon activation and that NKG2D–H60a interaction during cytotoxic T lymphocyte (CTL) differentiation reduces NOD CTL effector cytokine production (31).

## Function of NKG2D Ligand Expression by B Cells

Information regarding expression of NKG2D ligands by B cells is limited, and functional evidence is even scarcer. In humans, peripheral B cells have been shown to express ULBP1-3 (32). This study did not assess functional effects of ligand expression by these cells, but the authors did show that acute myeloid leukemia cells show very low NKG2D ligand expression compared to B cells from healthy patients, which may be a result of malignant transformation (32). Similarly, data from our laboratory demonstrate NKG2D ligand expression by B cells in aging mice (33). However, this expression was much greater in mice lacking NKG2D expression and correlated with increased B cell lymphoma development. This suggests that these NKG2D ligand-expressing B cells in older mice are premalignant, rather than healthy cells. A recent report hints that NKG2D ligand expression on B cells may play a role in cytokine production in Crohn’s disease. B cells in inflamed intestine were found to express ULPB1-6 and MICA/B, and a significant correlation between MICA/B expression by B cells and IL-1β and tumor necrosis factor alpha (TNF-α) production was demonstrated. In addition, a significant correlation was observed between ULBP expression by B cells and TNF-α release (30).

## Function of NKG2D Ligand Expression by NK Cells

With NKG2D being one of its major activating receptors, a large amount of NKG2D research has focused on NK cells. Despite this, relatively few studies have assessed the expression and function of NKG2D ligands on NK cells. In a 2003 study examining impaired NK cell function in NOD mice, Ogasawara and colleagues found that activated NK cells from NOD and NK1.1 NOD, but not C57BL/6 mice, express NKG2D ligands. RT-PCR analysis showed that these cells were positive for RAE-1α, β, and γ, and H60 mRNA transcripts. Only retinoic acid early inducible 1 (RAE-1) protein expression was confirmed by flow cytometry, as an anti-H60 antibody did not yet exist (34). The authors posit that expression of NKG2D ligands by NK cells themselves may contribute to the observed down modulation of NKG2D and NK cell dysfunction in NOD mice (34).

More recent studies suggest that expression of NKG2D ligands by NK cells may also direct NKG2D-mediated killing of NK cells, acting to regulate the NK cell response in a similar manner to that observed with T cells. The first of these studies describes the unique acquisition of tumor-derived RAE-1 by murine NK cells through the transfer of pieces of tumor membrane to NK cells in a process termed “trogocytosis.” These RAE-1-expressing NK cells were susceptible to NKG2D-dependent, perforin-mediated killing by other NK cells (35). Lopez-Cobo et al. also observed that human NK cells could acquire ULBP1-3 from target cells, which not only made them targets of autologous NK cell killing but also allowed for propagation of further NKG2D ligand transfer during this NK cell–NK cell interaction (36). In another study, type I interferons were shown to preserve NK cell expansion during murine cytomegalovirus infection by reducing NK cell expression of NKG2D ligands and reducing NKG2D-mediated fratricide (37).

Expression of NKG2D ligands by NK cells appears to have other functions besides targeting NK cells for killing. Brennan et al. observed expression of ULBP2 by stimulated human NK cells and found that expression was highest by recently activated and proliferating NK cells. This expression did not target NK cells for fratricide and led the authors to suggest that ULBP2 is a marker of newly activated “mature” NK cells (38). Recently, it was demonstrated in our laboratory that expression of ULBP proteins by activated NK cells plays an important role in tuning NK cells through regulation of TNF-α-converting enzyme (TACE) activity. We found that ULBP family members are upregulated on NK cells following activation with IL-12, IL-15, and IL-18. The interaction of NKG2D and NKG2D ligand, both expressed by NK cells, enhanced TACE activity, resulting in increased TNF-α and ULBP release from the cell surface (39).

## Function of NKG2D Ligand Expression by Monocytes and Macrophages

During the original characterization of MICA, Zwirner et al. found that MICA protein was expressed by monocytes from multiple donors using Western blot (19). Since, the expression of NKG2D ligands by monocytes and macrophages has been investigated by a number of groups, with results suggesting two primary functions. One of these functions was suggested by Hamerman et al., who showed that toll-like receptor (TLR) signaling through MyD88 in murine macrophages induced RAE-1 and that NK cells cocultured with these RAE-1-expressing macrophages internalized NKG2D from the surface both *in vitro* and *in vivo* (40). This suggests that ligand expression by macrophages is involved in communication between macrophages and NK cells. This idea is supported by another study showing that expression of RAE-1 by murine macrophages downregulates NKG2D surface expression by NK cells and inhibits the NK cell response against B16 tumors (41). MICA expression by human monocytes was also shown to enhance NK cell interferon gamma (IFN-γ) production and antitumor function *via* an NKG2D-dependent mechanism (42, 43).

In addition to communication with, and regulation of, NK cells, it appears that expression of NKG2D ligands also makes macrophages susceptible to regulation by direct NKG2D-mediated killing. Autologous killing of macrophages and monocytes by NK cells or NKG2D-expressing CD4^+^ T cells was shown after induction of NKG2D ligand expression on monocytes by lipopolysaccharide (LPS) stimulation, *in vitro* culture with IL-10, or on monocytes from patients with systemic lupus erythematosus (44–46). Other observations include upregulation of NKG2D ligands by human monocytes, as well as murine macrophages and microglial cells, in response to GM-CSF and other myeloid growth factors, including FLT-3 ligand and stem cell factor (32, 47). MICB and ULBP1 were also shown to be upregulated on healthy monocytes from glioblastoma patients in response to tumor-derived lactate dehydrogenase (48). Additionally, MICA and MICB were observed on foam cells from atherosclerotic lesions as well as human monocyte-derived macrophages treated with acetylated low-density lipoprotein, mimicking atherosclerotic conditions (49). A study by Ge et al. showed that while ULBP1 and MICA were expressed at similar levels by PBMC from children with Kawasaki disease and healthy controls, NKG2D expression by NK cells and CD8^+^ T cells was decreased in diseased patients, which correlated with increased cytokine production by monocytes (50). This observation suggests that NKG2D ligand expressed by monocytes (and possibly other PBMCs) interacting with NKG2D has functional effects in modulating monocyte function.

## Function of NKG2D Ligand Expression by Dendritic Cells (DCs)

Dendritic cells are cornerstones in initiating and directing an immune response. Given the evidence presented thus far of NKG2D ligand expression as a means of communication between immune cells, it is little surprise that NKG2D ligands are induced on DCs and appear to play a role in regulating DC, NK cell, and T cell function. In humans, ULBPs are not only upregulated after infection of DCs with influenza, measles, and respiratory syncytial virus but also after treatment with poly(I:C) (51, 52). Stimulation with LPS also induces ULPB2, MICA, and MICB, demonstrating danger signaling *via* TLRs can drive NKG2D ligand expression in DCs (42, 52).

Jinushi and colleagues described a pathway whereby IL-15 signaling drives expression of IFN-α, which induces MICA/B expression by monocyte-derived DCs (53, 54). They found that these DCs activated NK cells in a MICA/B-NKG2D interaction-dependent manner (54). The involvement of IL-15, a critical cytokine in NK cell development and activation, in inducing NKG2D ligand on DCs suggests the potentially important role of this ligand expression in the regulation of an NK cell-mediated response. It was later found that human DCs release exosomes bearing ULBP1 and IL-15 receptor and that these exosomes are capable of cross-presenting exogenous IL-15 to NK cells (55). Together, the presence of ULBP1 and IL-15 receptor by DC-derived exosomes promoted NK cell activation and proliferation (55). NKG2D ligands on DCs may be important regulators of T cell function as well. ULBP1 expression was observed by DCs in areas of T cell interaction in lymph nodes, suggesting a role for ULBP1 on DCs in the induction or reactivation of T cell responses (56). Zloza et al. found that transgenic expression of RAE-1ε on DCs at the time of priming rescued memory recall by CD8^+^ T cells in the absence of CD4^+^ T cells. They found that RAE-1 expression by DCs did not affect effector T cell responses, but conferred a high rate of survival of CD4^+^ T cell-deficient animals in a model of influenza in which viral elimination is ordinarily CD4^+^ T cell-dependent. Additionally, they showed that RAE-1 stimulation rescues HIV-specific CD8^+^ T cell responses in CD4^+^ T cell-deficient HIV-positive donors (57).

Evidence suggests that NKG2D ligand expression by DCs may not always be activating, but can also negatively regulate immune function. Transgenic expression of RAE-1ε by DCs causes downregulation of NKG2D on NK cells and impaired NKG2D-dependent NK cell functions, including tumor rejection (58). Correlative evidence comes from a study by Fabritius and colleagues who found expression of RAE-1γ on DCs in the spleen and lymph nodes of C57BL/6 mice and demonstrated that deletion of NKG2D accelerates rejection of cardiac allografts (59). Additionally, NKG2D ligand expression on DCs infected with an ULBP-expressing cytomegalovirus resulted in decreased MHC class I expression by the DCs (60). This effect is consistent with earlier studies from our laboratory that demonstrate that high NKG2D ligand expression decreases MHC class I expression by both tumor cells and normal cells (61). While this decrease in MHC class I increases NK cell responses, it likely decreases the response of CD8^+^ T cells (60, 61).

## Other Immune Cells

Many studies report expression of mRNAs encoding NKG2D ligands in both primary and secondary immune tissues (62–64). Much of this expression, especially in the thymus and spleen, is likely attributed to immune cell types already discussed. However, other immune cells have been found to express NKG2D ligands in both humans and mice, although the function of this expression is not entirely clear. RAE-1 and H60 are expressed by freshly isolated bone marrow cells from Balb/c, but not C57BL/6 mice, and this expression is responsible for the rejection of Balb/c bone marrow by C57BL/6 mice in an NKG2D-dependent manner (65). GR-1^+^CD11b^+^F4/80^+^ myeloid-derived suppressor cells (MDSCs) from RMA-S tumor-bearing mice were also found to express RAE-1. This expression enhanced the production of IFN-γ by NK cells and made the MDSCs susceptible to NK cell killing both *in vitro* and *in vivo* (66). Similarly, tumor-infiltrating myeloid cells in glioblastoma patients were shown to express MICB and ULBP1 (48). It has yet to be determined if expression of NKG2D ligands in these instances is an incidental effect of rapid cellular division in bone marrow or immune dysregulation in the tumor environment, or if they play a distinct role in immune cell development and regulation. However, given the cellular energy involved in protein expression, and the potential immune triggering consequences, it seems unlikely that NKG2D ligands would be induced without a biologically important function.

## Conclusion

**Figure 1:**
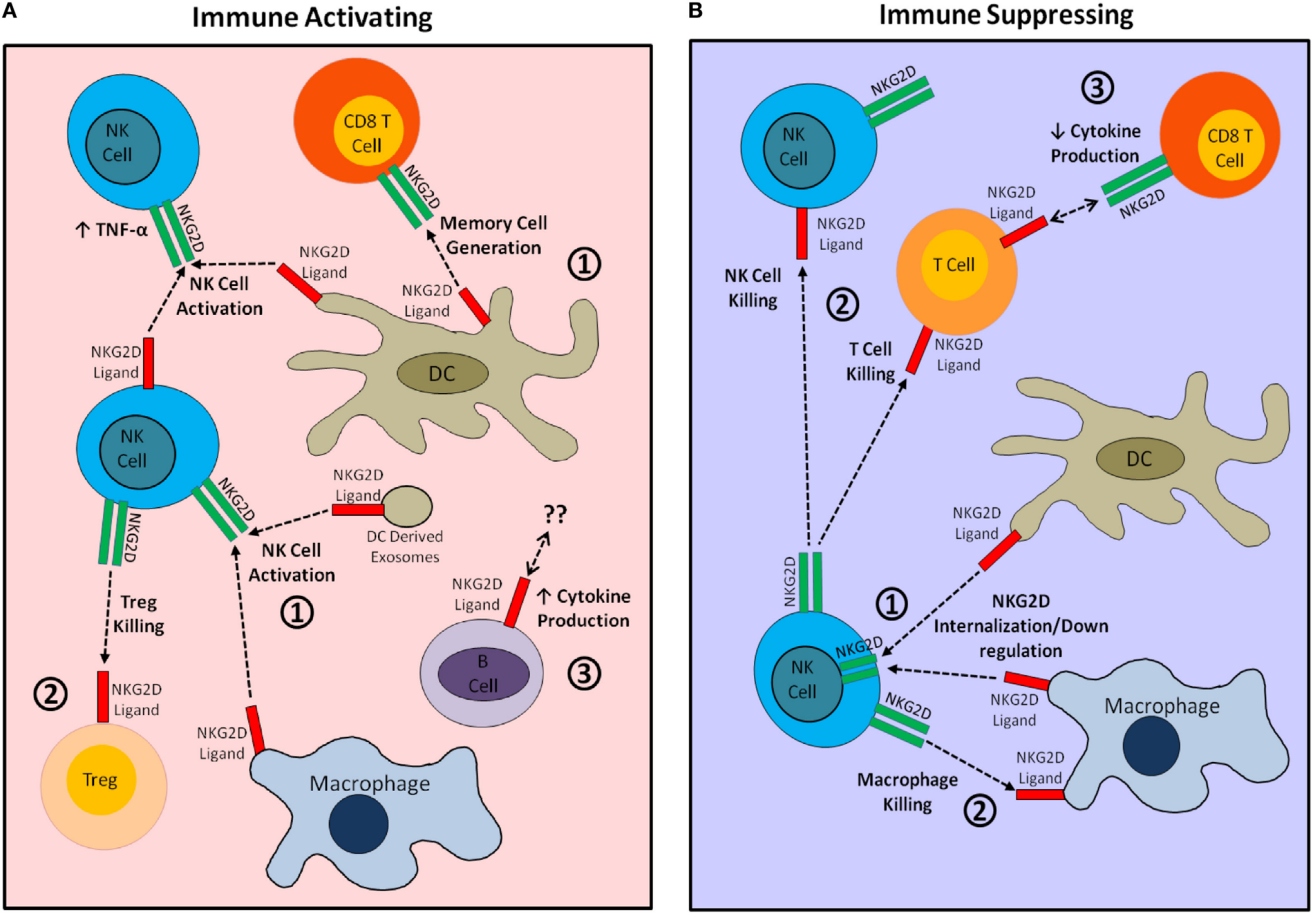
Visual summary of immunostimulatory and immunosuppressive effects of natural killer group 2 member D (NKG2D) ligand expression by cells of the immune system. **(A)** The immunostimulatory effects of NKG2D ligand expression by immune cells. (1) NKG2D ligand expression by dendritic cells (DCs) and macrophages provides activating and differentiation signals to NKG2D-bearing natural killer (NK) cells and CD8^+^ T cells. (2) Expression of NKG2D ligand by regulatory T cells (Tregs) targets these cells for killing by NK cells, thereby increasing the overall immune response. (3) NKG2D ligand expression may affect B cell cytokine production. **(B)** The immunosuppressive effects of NKG2D ligand expression by immune cells. (1) Widespread expression of NKG2D ligands by DCs or macrophages causes internalization of NKG2D, impairing NKG2D-mediated immune activation. (2) NKG2D ligand-bearing immune cells are directly targeted for killing by autologous NKG2D-bearing NK and T cells. (3) NKG2D–ligand interaction during CTL generation may decrease CTL cytokine production.

The effects of NKG2D ligand expression by immune cells are summarized in Figure 1 and Tables 1 and 2. A major theme is that expression of NKG2D ligands by immune cells serves to downregulate immune responses. This is done either by modulating the activity of other NKG2D-bearing cells or making the ligand-bearing cells susceptible to NKG2D-mediated killing. A unique aspect of NKG2D ligand expression is the paradoxical ability of these ligands to not only stimulate a robust immune killing response in some instances but also exhibit expression on a diverse array of healthy tissues. This can be explained in large part by the expression of inhibitory “self” molecules by healthy cells, which limit or prevent their killing (67–70). Interestingly, animals engineered with ubiquitous transgenic expression of NKG2D ligands show surprisingly understated effects on immune development and response. With the exception of an early report, which found weight loss, hyperkeratosis, and increased white blood cell numbers in mice ubiquitously expressing MICB (71), studies involving transgenic expression of MICA, RAE-1, or H60a have shown effects limited to downregulation of NKG2D expression, impaired NKG2D-dependent NK cell and CD8^+^ T cell functions, and decreased MHC class I expression (15, 61, 65, 72–76), with limited effects on immune responses. Such a finding does not preclude the critical role NKG2D may play in immune function and regulation. As we have seen, NKG2D ligand expression by immune cells appears to play opposing roles in regulating immune responses. In some instances, ligand expression regulates immune cell responses by targeting the ligand-bearing cell for NKG2D-mediated killing. NKG2D ligand expressing immune cells can also reduce NKG2D-dependent immunity by directly causing internalization of NKG2D itself. In other cases, NKG2D ligands expressed by immune cells stimulate the activation and proliferation of NKG2D-positive cells without necessarily inducing killing against the ligand-bearing cell. In addition, NKG2D ligands on non-immune cells can recruit immune cells to the site of expression (17, 30). It is likely that NKG2D ligand expression by immune cells similarly acts to recruit immune cells and enhance a local immune response. Which of these immune modulatory effects of NKG2D ligand expression prevails is likely situational, depending upon the combination of many factors present, and could be a key component in maintaining an effective yet measured immune response. Yet another compelling reason to better understand the function of NKG2D ligand expression by immune cells comes from the wide interest in targeting NKG2D ligand expression in both cancer and autoimmunity. Such an understanding will help avoid side effects and improve the efficacy of therapies such as NKG2D chimeric antigen receptor T cells, which are being developed to target tumors, and anti-NKG2D mAbs, which are being evaluated for the treatment of Crohn’s disease and type I diabetes (77–81).

**Table 1:** Summary of natural killer group 2 member D (NKG2D) ligands expressed by human immune cells and their observed immunological effects.

Immune cell type	Observed NKG2D ligands	Immune activating effects	Immune suppressing effects	Reference
T cells	Major histocompatibility complex (MHC) class I polypeptide-related sequence A (MICA) MHC class I polypeptide-related sequence B (MICB) ULBP1-3	Enhanced natural killer (NK) cell activation NK cell killing of regulatory T cells	NK cell killing of T cells Downregulation of NKG2D through release of soluble ligands	(19–21, 24–27, 29)

B cells	ULBP1-6 MICA MICB	Correlation with increased IL-1β and tumor necrosis factor alpha (TNF-α) production	–	(30, 32)

NK cells	ULBP1-6	Increased TNF-α release	NK cell–NK cell killing	(36, 38, 39)

Monocytes/macrophages	MICA MICB ULBP1-3	Increased NK cell interferon gamma production	Macrophage/monocyte killing	(19, 42–46)

Dendritic cells	MICA MICB ULBP1-3,5	Activation of NK cells NKG2D ligand bearing exosomes promote NK cell activation and proliferation	–	(51, 52, 54–56)

**Table 2:** Summary of natural killer group 2 member D (NKG2D) ligands expressed by immune cells in mice and their observed immunological effects.

Immune cell type	Observed NKG2D ligands	Immune activating effects	Immune suppressing effects	Reference
T cells	H60a H60b* murine ULBP-like transcript 1 (MULT1)*	–	Natural killer (NK) cell killing of T cells Decreased CD8^+^ T cell cytokine production	(22, 23, 31)

B cells	Retinoic acid early inducible 1 (RAE-1) MULT1	Protection from B cell lymphoma		(33)

NK cells	RAE-1α-γ H60*	–	NK cell–NK cell killing	(34, 35, 37)

Monocytes/macrophages	RAE-1	Correlation with microglial proliferation	Downregulation of NKG2D on NK cells	(40, 41, 47)

Dendritic cells	RAE-1	Enhanced CD8^+^ T cell memory generation in the absence of CD4^+^ T cells	Downregulation of NKG2D on NK cells and impaired NK cell function Decreased major histocompatibility complex-I expression	(57, 58, 60, 61)

Myeloid-derived suppressor cells	RAE-1	Increased NK cell IFNγ production and killing of MDSCs	–	(66)

*Observed at the mRNA level only.

Finally, evidence is accumulating that NKG2D–NKG2D ligand interaction between immune cells has functions beyond a stimulatory capacity, including T cell development, differentiation, and memory generation. So far, we only have a glimpse of how NKG2D signaling affects immune cells in these ways. Another major unresolved question is whether the NKG2D ligands are functionally different. There are hints that this may be the case, though evidence suggests that differences may be driven more by ligand tethering, GPI-anchor versus transmembrane domain, which affects distribution on the cell surface, and physical size, than by the affinity for or interaction with individual ligands for the NKG2D receptor itself (82, 83). Further research in these areas may shed light on the complex and sometimes conflicting roles for NKG2D reported in disease and tumor immunity. In summary, it is apparent that NKG2D ligand expression by immune cells themselves warrants more rigorous investigation as a multifaceted mechanism regulating immune system function.

## Author Contributions

AT wrote the majority of the manuscript. MM contributed to writing and editing the manuscript.

## Conflict of Interest Statement

The authors declare that the research was conducted in the absence of any commercial or financial relationships that could be construed as a potential conflict of interest.

## Abbreviations

ATM, ataxia telangiectasia mutated; ATR, ataxia telangiectasia mutated- and Rad3-related protein; CAR, chimeric antigen receptor; CMV, cytomegalovirus; CTL, cytotoxic T lymphocyte; DAP10, DNAX-activating protein of 10 kDa; DAP12, DNAX-activating protein of 12 kDa; IFN-γ, interferon gamma; *Klrk1*, killer cell lectin-like receptor K1; LPS, lipopolysaccharide; MCMV, murine cytomegalovirus; MDSCs, myeloid-derived suppressor cells; MHC, major histocompatibility complex; MICA, MHC class I polypeptide-related sequence A; MICB, MHC class I polypeptide-related sequence B; MULT1, murine ULBP-like transcript 1; NKG2D, natural killer group 2 member D; NOD, non-obese diabetic; PBMC, peripheral blood mononuclear cell; PHA, phytohemagglutinin; RAE-1, retinoic acid early inducible 1; RAET1, retinoic acid early transcript 1; TACE, TNF-α-converting enzyme; TNF-α, tumor necrosis factor alpha; ULBP, UL16-binding proteins.
